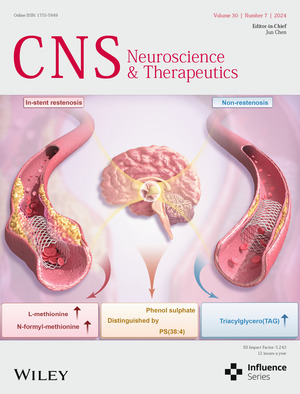# Front Cover

**DOI:** 10.1111/cns.14898

**Published:** 2024-08-02

## Abstract

Cover image: The cover image is based on the article *Alterations of Metabolome and Lipidome in Patients with In‐Stent Restenosis* by Ziqi Xu et al., https://doi.org/10.1111/cns.14832.